# Prenatal detection of a giant isolated coronary fistula

**DOI:** 10.1002/ccr3.3779

**Published:** 2021-02-04

**Authors:** Adeline Walter, Elina Calite, Alexander C. Engels, Ulrike Herberg, Johannes Breuer, Arne Willruth, Christoph Berg, Annegret Geipel, Ulrich Gembruch

**Affiliations:** ^1^ Department of Obstetrics and Prenatal Medicine University Hospital Bonn Bonn Germany; ^2^ Department of Pediatric Cardiology University Hospital Bonn Bonn Germany; ^3^ Department of Obstetrics and Prenatal Medicine University Hospital Cologne Cologne Germany

**Keywords:** congenital heart diseases, coronary artery, fetal heart, prenatal detection, prenatal heart failure, prenatal high cardiac output

## Abstract

Prenatal detection of an isolated congenital coronary artery fistula (ICCAF) requires an examination of the affected fetal hemodynamic situation by the fistula. Early pediatric cardiological presentation is needed, since prenatal changes may have relevant postpartal consequences.

## Graphical abstract

Prenatal detection of an ICCAF requires an examination of the affected fetal hemodynamic situation by the fistula. Early pediatric cardiological presentation is needed, since prenatal changes may have relevant postpartal consequences.

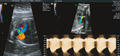

## 1 INTRODUCTION

Isolated congenital coronary artery fistula (ICCAF) is a rare malformation that counts for 0.2% to 0.4% of all congenital heart diseases.^1^ It is defined as an abnormal connection of a coronary artery with one of the four cardiac chambers or any vessel.^2^ As data are limited and as structured evaluation of fetal coronary arteries is not regularly performed, knowledge about possible prenatal prognostic parameters and prenatal consultation, if detected, is rare. Fetuses might be at risk to develop a high cardiac output failure, which might be developed soon after birth and require an immediate operative intervention.

We describe a case of the largest prenatally diagnosed ICCAF draining into the right atrium, with the highest measured Doppler velocity and the most pronounced fetal cardiomegaly among the previously published once. Cardiomegaly did not correlate with a worsen outcome.

## 2 CASE

**FIGURE 1:**
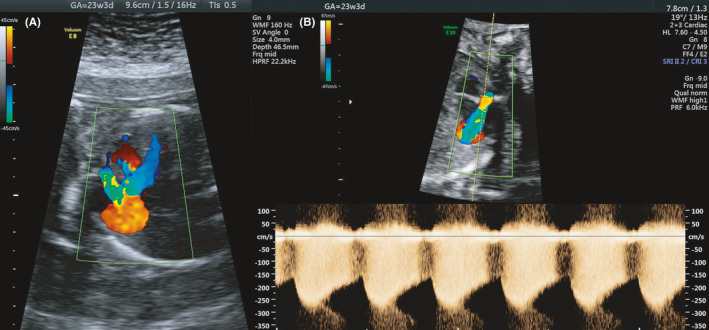
Color Doppler examination at 23 + 3 weeks of gestation showing the turbulent flow in the coronary artery fistula originating from the RCA draining to the right atrium (A). Continuous wave Doppler measuring maximum velocity of 3.2 m/s in the coronary fistula (B)

A 36‐year‐old woman, gravida 8 para 3, was referred to our department at 22 + 3 weeks of gestation because of suspected cardiac anomaly. Fetal echocardiography showed a situs solitus with concordant atrio‐ventricular and ventriculo‐arterial connections as well as morphologically normal heart valves. On detailed examination, right atrium and main pulmonary artery (5.6 mm, normal: 3.3 ‐ 4.6 mm) were noted to be slightly enlarged. Color flow imaging demonstrated a turbulent jet lasting the whole cardiac cycle into the right atrium with maximum velocities of 3.2 m/s (Figure 1). The origin of the jet was detected to be near the ascending aorta in the region of the right coronary artery (RCA), having a vascular connection to the right atrium. As no other abnormalities were detected, diagnosis of an isolated right coronary artery to right atrium fistula of 9 mm in size was made.

**FIGURE 2:**
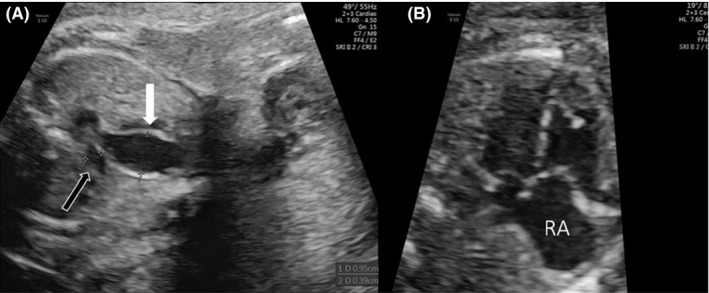
Ultrasound examination at 27 + 5 weeks of gestation showing the severely dilated pulmonary artery of 9.5 mm in size (filled arrow) in comparison with the ascending aorta (continuous arrow) of 3.9 mm in size (A). Four‐chamber view demonstrating the dilated right atrium (RA) at 23 + 3 weeks of gestation (B)

Follow‐up examinations showed further dilatation of the right atrium and the main pulmonary artery (9.5 mm) (Figure 2). At 27 + 5 weeks, cardiomegaly (cardiothoracic area ratio, CTAR: 0.659) and a to‐and‐fro flow of the aortic arch resulting from the steal effect across the coronary fistula were observed the first time (Movie S1 in the Data Supplement). At 33 + 3 weeks, increased flow velocity in the fistula to 3.7 m/s and one week later increased pulsatility of the flow velocity waveforms in the umbilical artery were measured. Vaginal delivery was planned for 36 + 3 weeks. After induction with misoprostol, a vaginal bleeding occurred and an urgent Cesarean section was performed. A 2,560‐g male fetus with Apgar scores of 5, 9, and 9 at 1, 5, and 10 minutes, respectively, was delivered.

Postnatal echocardiography confirmed prenatal findings (Movie S2 in the Data Supplement). The RCA was massively dilated with a maximal diameter of 10.4 mm at its aortic origin, becoming smaller after a course of 16 mm with a minimal diameter of 1.45 mm at the right atrial communication. There was a continuous systolic and early to mid‐diastolic run off from the aorta into the right atrium with a gradient of 3.8 m/s at the atrial level. In addition a small muscular ventricular septal defect, a dilated ascending aorta with a narrow aortic arch and a patent arterial duct were also detected. The aortic changes were interpreted as hemodynamic response to the intrauterine steal effect via the fistula. On the fifth day of life, a cardiac catheterization procedure was performed and an Amplatzer duct occluder II (4 x 4) was successfully placed in the proximal RCA (Movie S3 in the Data Supplement). The RCA was noted to be interrupted with a complete confluence of the fistula to the right atrium. The distal part of the coronary artery was perfused retrogradely via the left coronary artery. Unfortunately, the narrow aortic arch progressed to a coarctation of the aorta (CoA) with a blood flow velocity of 4 m/s after ductal occlusion. Surgical resection and end‐to‐end anastomosis was performed at 22 days of life. Six months later, a restenosis of the resected area occurred and balloon angioplasty was performed. The baby is now one year old.

## 3 DISCUSSION

Depending on the structures connected by the fistula and its size, a significant hemodynamic change may occur during fetal period.^3^ In particular, a connection of a high pressure to a low pressure system, as seen in coronary artery to atrium fistulas, is crucial.^4^, ^5^ If a relevant aortic to atrial shunt through the fistula is present, a turbulent jet lasting the whole cardiac cycle and a dilatation of the affected feeding coronary artery are typically seen prenatally. In addition, cardiac chamber enlargement or possible fetal hydrops due to high cardiac output failure may also be detected.^3^, ^6^ These fetuses are at risk of heart failure progressing soon after birth as peripheral vascular resistance, consequently cardiac workload and left‐to‐right shunting through the fistula increase. Postnatal coronary perfusion competes with system perfusion, especially if ductus occlusion has failed. In these patients, operative intervention might be required during the neonatal period, although in most cases later interventional treatment is sufficient.^4^, ^7^

Prenatal evaluation of coronary anatomy and circulation is extremely difficult due to the small vessel size and the needed examiner expertise.^8^, ^9^ Evaluation of them can be primary achieved by color flow imaging and pulsed waved Doppler. Main coronary arteries are best analyzed in a lateral long‐axis view, or a modified short‐axis view of the left ventricular outflow tract and ascending aorta. Color Doppler velocity scale should be adjusted between 0.3 and 0.7 m/s. The sample volume for pulsed wave Doppler should be adapted to exclude other cardiac and extracardiac blood flows, the high‐pass filter set at 100 ‐ 125 Hz, and the insonation angle kept as close to 0° as possible.^10^

Nevertheless, ICCAF’s may be mistaken for ventricular septal defects or valve regurgitation, or even be undetected if flow is too small or at low velocity, especially when using too high pulse repetition frequencies.^11^

If prenatally detected, connected structures must be identified to estimate the hemodynamic situation. A to‐and‐fro flow of the aortic arch resulting from the steal effect across the coronary fistula must be associated with a huge blood volume flow in the fistula. This leads to the risk of high cardiac output on one side and to a possible postnatal risk of developing a CoA due to a prenatally reduced flow through the aortic arch on the other side.

Furthermore, prenatal presence of cardiomegaly and the distance between the main coronary artery and the atrium should be evaluated, as they seem to be good parameters for assessing postnatal heart failure.^12^

## 4 CONCLUSION

Prenatal detection of ICCAF’s is desirable as it might change management and outcome in affected patients. Evaluating the prenatal hemodynamic situation by determining the connected structures should be of major importance, as they may lead to fetal and long‐term health defects. If prenatally observed, follow‐up examinations should be carried out to identify possible progression and to time delivery and therapeutic intervention, if needed. The examiner should pay particular attention to a presence of a cardiomegaly, cardiac chamber enlargement, and a to‐and‐fro flow in the aortic arch, resulting from the steal effect across the coronary fistula which must be associated with a huge blood volume flow in the fistula. Delivery should be inducted, if hydrops fetalis or a centralization of fetal blood flow caused by ICCAF is prenatally observed. Further depending on gestational age, occurrence of a significant atrioventricular valve insufficiency, an increased pulsatility index of the umbilical artery, resulting from the rising steal effect through the fistula are secondary signs of a beginning heart failure that might be considered as further indications for delivery.

Delivery should take place in a perinatal center with a pediatric cardiology unit.

Nevertheless, further investigations are needed to evaluate other possible prognostic parameters and to get more knowledge about the pathophysiology of this disease.

In conclusion, our case describes the largest isolated fistula to the right atrium, with the highest measured Doppler velocity and the most pronounced fetal cardiomegaly among the previously published ICCAFs. Severe valve insufficiencies or a fetal hydrops did not occur. Prenatal detection made an early intervention possible, leading to a good clinical outcome.

## CONFLICT OF INTEREST

There are no conflicts of interest to be declared.

## AUTHOR CONTRIBUTIONS

UH, AW, CB, AG, and UG: managed the patient. AW, EC, UH, JB, AG, and UG: performed the analysis. AW and ACE: created the figures. All the authors: contributed in writing and editing of the manuscript.

## ETHICAL APPROVAL

Written informed consent was obtained from the patient for the publication.

## Supporting information

Video S1

Video S2

Video S3

Supplementary Material

## Data Availability

The data that support the findings of this case are available from the corresponding author upon reasonable request.
