# Supplementary material for: Prenatal detection of a giant isolated coronary fistula
Source: Clin Case Rep. 2021 Feb 4;9(3):1393–6. doi: 10.1002/ccr3.3779 (PMC7981625; doi:10.1002/ccr3.3779)
Supplement: Supplementary file 4 — Supplementary Material [file CCR3-9-1393-s001.docx]

**Supplemental Material** (online data supplement)

**Movie I** Color Doppler examination at 27+5 weeks of gestation showing a to-and-fro flow of the aortic arch resulting from the steal effect across the coronary fistula and the turbulent jet in the coronary artery fistula originating from the right coronary artery (RCA) draining to the right atrium.

**Movie II** Postnatal echocardiography at the first day of life showing the coronary artery fistula.

**Movie III** Ultrasound examination on the fifth day of life demonstrating the successfully placed Amplatzer duct occluder II in the proximal RCA.
